# Lightweight dynamic model for fusion of three-dimensional surface–stratum underground structures

**DOI:** 10.1371/journal.pone.0326167

**Published:** 2025-06-26

**Authors:** Cuiying Zhou, Yusen Zhong, Ziyu Tao, Chunhui Lan, Wei Hu, Zhen Liu

**Affiliations:** 1 School of Civil and Transportation Engineering, Guangdong University of Technology, Guangzhou, China; 2 Guangdong Engineering Research Centre for Major Infrastructure Safety, Guangzhou, China; 3 School of Civil Engineering, Sun Yat-sen University, Guangzhou, China; Sichuan University of Science and Engineering, CHINA

## Abstract

Three-dimensional visual modeling of surface-stratum-structure is an inevitable requirement for intelligent geotechnical engineering. However, the heterogeneity of surface, stratigraphic and structural data sources and modeling methods leads to incompatibility of model attributes. Only a few studies have directly realized the three-dimensional (3D) modeling of surface-strata-structure. In order to solve this problem, this study uses the Kriging interpolation method based on relative elevation to realize the fusion of borehole data and surface data, and establishes a 3D surface-strata model. The Boolean difference set operation based on the bounding box method is used to propose a geometric fusion method for the 3D surface-strata-underground structure model. In addition, an attribute fusion method of 3D surface-stratum-underground structure model is introduced by using variable storage method. Finally, this study established a comprehensive 3D model of surface-strata-underground structure. Considering the characteristics of the fusion model, the dynamic expression of the 3D settlement model is realized, and a lightweight processing method using layered rendering technology is proposed, which is helpful to establish a lightweight 3D dynamic model of surface-strata-underground structure. The feasibility and reliability of the above method are verified by engineering application analysis. The main contributions of this method include simplifying the integration process of surface, stratum and structure models, which may enhance the modeling of integrated engineering information.

## Introduction

Three-dimensional (3D) visual modeling is imperative as it facilitates the intuitive and lucid presentation of all aspects of infrastructure and geological bodies, thus allowing enhanced comprehension and management. The visualization of infrastructures and geological bodies is contingent upon the availability of accurate dynamic models of surface, stratum, and engineering structures. However, significant discrepancies exist among the data sources and modeling methods employed in the development of 3D surface, 3D stratigraphic, and building models, resulting in their development as distinct entities, with limited emphasis on dynamic representation. The integration and dynamic expression of surface, stratigraphic, and structural models offer substantial benefits to academia and infrastructure engineering.

Current studies in surface, stratigraphic, and structural modeling typically involve distinct processes. Surface and stratigraphic models are collectively referred to as 3D geological models [1]. Currently, the most common 3D geological modeling methods can be classified into four categories: modeling methods based on borehole data [2–5], parallel sections, mesh cross-sections [6–10] and multisource interactive modeling [11,12]. The development of satellite remote-sensing technology [13–16], laser scanning technology, and aerial photography [17–20] has contributed to the expansion of ground surface data for 3D surface modeling, making it highly accurate and easily obtainable [21–24].The primary data sources for 3D stratigraphic modeling encompass borehole data, commonly used planar geological maps, 3D grid data, cross-folding profiles, and other underground data. These data sources are characterized by high acquisition cost and a level of accuracy that typically falls short of that achieved with surface data. Therefore, in current 3D geological modeling practices, surface and stratigraphic data are typically managed separately, with separate surface and stratigraphic models being established accordingly. Some scholars have also studied the overall modeling by establishing the feature unwrapping theory under the spatial constraints of the geological field and the interpretation system based on the large language model [25–27]. The modeling of underground structures relies primarily on information modeling technology [28,29].Many scholars have improved the efficiency of each stage of the project based on information technology optimization [30–33], which has become an industry standard widely implemented in Europe and the United States [34]. Many scholars also rely on information technology to achieve a variety of ways of 3D modeling, greatly improving the efficiency of modeling and project management efficiency [35–37].However, structural models constructed using information technology include a diverse array of information beyond mere geometric information [38,39]. Additionally, the file formats of these models are often incompatible with those of surface and stratigraphic models [40]; Thus, they cannot be directly integrated with surface and stratigraphic models. Extensive studies have been conducted on the separate modeling of surfaces, stratigraphy, and structures. However, there is a paucity of research on the fusion modeling of these components and the dynamic expression of the resulting integrated model. First, in studies regarding the fusion of surface and stratigraphic models, surface and subsurface data are generally considered as disparate source data. For example, researcher [41]proposed a method based on the fusion of multisource data and a 3D stratigraphic modeling approach, leveraging the fusion results. However, they used the absolute elevation of the data for spatial interpolation, which resulted in varying degrees of model smoothness between the surface and subsurface sections owing to the differing accuracies of the surface and subsurface data. Second, the majority of studies concerning the fusion of structural and stratigraphic models focus on the integration of underground structural models and surface models [42–47]. The fundamental objective is to load the structural information model into a Geographic Information System (GIS) platform through data conversion to fuse the information and GIS models [48]. However, this approach merely realizes attribute fusion between models within their spatial dimensions, i.e., the subsurface model is disregarded, thereby omitting consideration of the geometric inconsistencies between the models.

Therefore, to address the challenge of integrating surface, stratigraphic, and underground structural models, we propose the Kriging-interpolation method tailored to account for relative elevation, adopt the Boolean-difference operation grounded in the enveloping-box method, and incorporate the variable-storage method to achieve the seamless integration of geometric and attribute data. This integrated approach is expected to achieve the construction of a 3D surface–stratigraphic–underground structure model. Subsequently, based on the characteristics of the fused model, its dynamic expression is derived, and lightweight loading is performed using hierarchical rendering techniques.

## Research content and methodology

**Fig 1:**
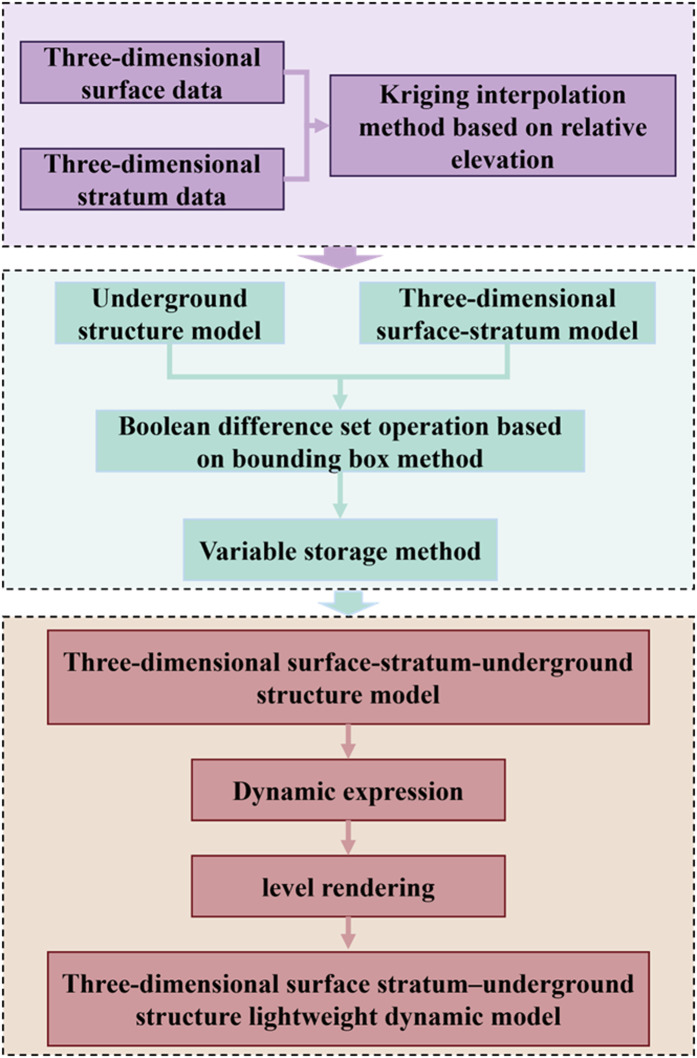
Technical route.

First, we perform the fusion modeling of 3D surface and stratum models through the application of Kriging interpolation based on relative elevation data. Second, we proceed with the fusion modeling of 3D surface–stratum–subterranean structure by executing Boolean-difference operations that leverage the enveloping-box and variable-storage methodologies. Subsequently, we derive the dynamic expression based on the distinctive features of the fusion model. Ultimately, we achieve lightweight loading employing the hierarchical rendering technique. The adopted technical route is shown in Fig 1.

### Kriging interpolation for 3D surface–stratigraphic models based on relative elevation

**Fig 2:**
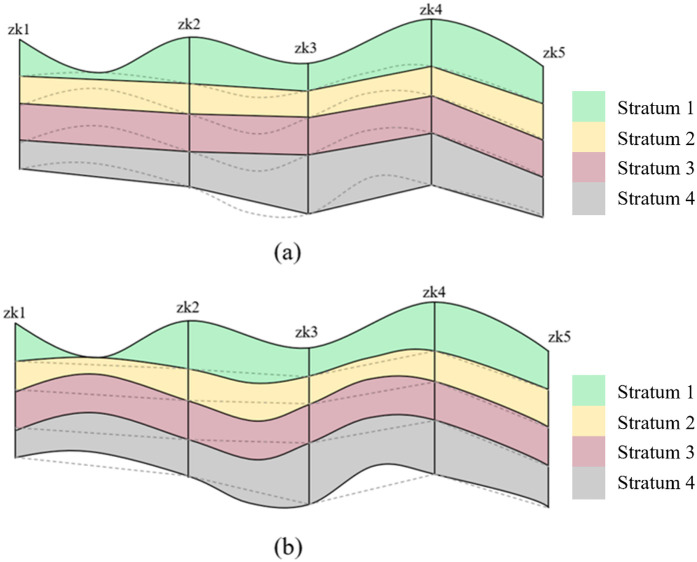
3D surface–stratigraphic model.

In 3D stratigraphic modeling, the process of interpolation calculation entails the interpolation of the absolute elevation of each stratum at the interpolation point to derive the absolute elevation. Subsequently, a 3D stratigraphic model is constructed by connecting and aligning the interpolation points. This interpolation method, which is based on the absolute elevation, does not leverage the high accuracy of 3D surface data. Consequently, the 3D stratigraphic model constructed based on a limited amount of borehole data is typically not smooth and fails to accurately reflect the actual situation (as shown in Fig 2(a)):

Hence, this study leverages the advantages of 3D surface data, associates it with 3D stratigraphic data, assigns relevant stratigraphic information to the surface data, and classifies the surface data based on different stratigraphic information. The approach also involves adopting the relative elevation of the stratum to be interpolated and its preceding stratum as the attribute value of Kriging interpolation, ensuring that each stratum is associated with its preceding stratigraphic data. Subsequently, Kriging interpolation is performed to construct a 3D surface–stratigraphy model, as shown in Fig 2(b).

In comparison to the results derived from direct interpolation using absolute elevation, the utilization of interpolation based on relative elevation exhibits superior spatial consistency. Consequently, the resultant 3D surface–stratigraphy model demonstrates enhanced smoothness and coherence, both horizontally and vertically. Furthermore, the model can effectively avoid erroneous connectivity within the surface layer, thereby ensuring more reliable interpolation calculation results.

The establishment of the 3D surface–stratigraphic model using the relative elevation-based Kriging interpolation method involves the following key aspects: calculating the absolute elevation of the surface using the Kriging method to yield an absolute-elevation dataset of the surface based on 3D surface data, calculating the elevation of each stratum relative to the surface using the Kriging method to yield a relative-elevation dataset of each stratum based on 3D stratigraphic data, and integrating the absolute-elevation datasets of the surface and strata to fit the 3D surface–stratigraphic fusion model. The specific steps involved in this process are as follows:

(1) Determine the uniform raster grid, Grid0, and perform 3D surface discrete point interpolation calculations.

**Fig 3:**
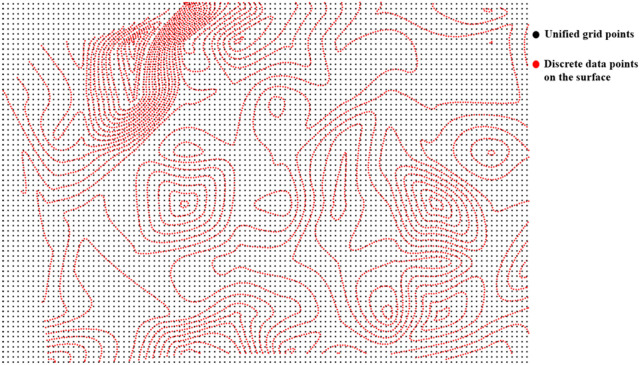
Schematic diagram of unified raster grid Grid_0_ and discrete data points on surface.

Given the accessibility and precision of surface data relative to underground borehole data, surface information derived from borehole data can be disregarded during the surface model fitting process. For the 3D surface data, the absolute elevation is employed as the attribute value. A uniform raster grid, Grid_0_, is determined, and the raster points are utilized as interpolation points to perform Kriging-interpolation calculation, thereby yielding the absolute-elevation data of the surface, which are recorded as SurfData, as illustrated in Fig 3.

(2) Processing of surface absolute-elevation data.

Surface absolute-elevation data generally contain only spatial geographic information, precluding association with subsurface information. Consequently, classification and extraction of features, identification of the lithology, and integration of geomorphological and lithological information into the surface absolute-elevation data are essential. The lithology is primarily identified through a comparative analysis of high-resolution remote-sensing images with the surface absolute-elevation data, followed by the assignment of lithological information to the surface absolute-elevation data based on regional characteristics. Furthermore, it can be identified through the observation of morphology, color, texture, and other characteristics of the local surface, supported by detailed geological information provided in geological survey reports and on-site photographs. Following this, a comprehensive lithography assessment is conducted, culminating in the assignment of lithological information to the local counterpart of the surface. Assuming that the surface–stratigraphy study area is designated as A, the surface absolute-elevation data are partitioned into areas based on stratigraphy, with this division informed by lithological information and underground stratigraphy categories, as follows:


$$\begin{array}{c} \begin{array}{c} A\left\{ \;\;\;\begin{array}{c} {layer}_{0}\\ {layer}_{1}\\ \begin{array}{c} \vdots \\ {layer}_{n} \end{array} \end{array}\right.\; \end{array} \end{array}$$ (1)

In Equation 1, layer0 represents the ground surface, layer_1_ represents the stratum with stratigraphic sequence number 1, and this numbering convention continues sequentially; and layer_n_ represents the stratum with stratigraphic sequence number n. The surface absolute-elevation data are categorized by the geographical regions in which they are situated, i.e.,


$$\begin{array}{c} SurfData\left\{ \begin{array}{c} H_{0}(x)\\ H_{1}(x)\\ \begin{array}{c} \vdots \\ H_{n}(x) \end{array} \end{array}\right.\; \end{array}$$ (2)

In Equation 2, H_0_ represents the area of layer_0_; SurfData[H_0_] represents the elevation of points within this area, i.e., the surface elevation; and H_1_ represents the elevation at points within layer_1_, i.e., the bottom elevation of the stratum point with sequence 1, and this pattern continues for subsequent layers. In general, the surface point data of SurfData are distributed centrally within layer_0_ and locally distributed in layer_1_, layer_2_, and so forth.

(3) Stratigraphy layer_1_ (stratum with stratigraphic sequence number—relative elevation interpolation calculation).

Based on the 3D stratigraphic data model, the bottom elevation of layer_1_ is denoted as ZKData[H_1_(x)], and the relative elevation within layer_1_ is written as


$$\begin{array}{c} \begin{array}{c} \left\{ \;\;\;\begin{array}{c} h_{1}(x)=SurfData\left[H_{0}(x)\right]-ZKData\left[H_{1}(x)\right],x\in {layer}_{0}\\ h_{1}(x)=0,x\notin {layer}_{0} \end{array}\right.\; \end{array} \end{array}$$ (3)

The relative elevations of layer_1_ are expressed relative to the ground surface and serve as attribute values for Kriging interpolation to ensure the computability of the relative elevations. Meanwhile, interpolation calculations are executed in the uniform raster grid Grid_0_ to obtain the relative-elevation data corresponding to stratigraphic sequence number 1, denoted as h_1_Data(x).

(4) Calculation of relative-elevation interpolation for each stratum sequence.

The bottom elevation of the layer for collating all the borehole data is denoted as ZKData[H_n_(x)], where n is the stratigraphic sequence number. Therefore, the relative elevation of the stratum in layer_n_ is written as:


$$\begin{array}{c} \begin{array}{c} \left\{ \;\;\;\begin{array}{c} h_{n}(x)=ZKData\left[H_{n-1}(x)\right]-ZKData\left[H_{n}(x)\right],x\in {layer}_{0\sim n-2}\\ \begin{array}{cc} h_{n}(x)=SurfData\left[H_{n-1}(x)\right]-ZKData\left[H_{n}(x)\right],x\in {layer}_{n-1} & \\ h_{n}(x)=0,x\notin {layer}_{0\sim n-1} \end{array} \end{array}\right.\; \end{array} \end{array}$$ (4)

Finally, the relative-elevation data of each stratum sequence are obtained by assigning each relative elevation as an attribute value within the uniform raster grid Grid_0_ for performing Kriging-interpolation calculation. The relative-elevation data for each stratum sequence are obtained by assigning each relative elevation as an attribute value for Kriging interpolation.

The integration of the 3D stratigraphic and surface data facilitates the acquisition of the relative elevation data for each sequence of stratigraphic layers via Kriging interpolation based on the relative elevation. The establishment of the 3D surface–stratigraphic fusion model hinges upon the identification of the relationship between the stratigraphic relative-elevation data and the surface absolute-elevation data, which is essential for deriving the absolute elevation data for each sequence of the stratigraphic layers.

The surface absolute-elevation data SurfData and relative-elevation data of each stratigraphic sequence are calculated based on the unified raster Grid_0_, and therefore, they can be directly superimposed.

The surface absolute elevation is written as:


$$\begin{array}{c} H_{0}Data(x)=SurfData\left[H_{0}(x)\right] \end{array}$$ (5)

The obtained H_0_ Data(x) is the elevation of the top of the stratum in layer_1_.

The absolute elevation of the stratum in layer_n_ is written as:


$$\begin{array}{c} \begin{array}{c} \left\{ \;\;\;\begin{array}{c} H_{n}Data(x)=H_{n-1}Data(x)-h_{n}Data(x),x\in {layer}_{0\sim n-1}\\ H_{n}Data(x)=SurfData\left[H_{n}(x)\right],x\notin {layer}_{0\sim n-1} \end{array}\right.\; \end{array} \end{array}$$ (6)

The obtained H_n_ Data(x) is also the stratum layer_n_.

**Fig 4:**
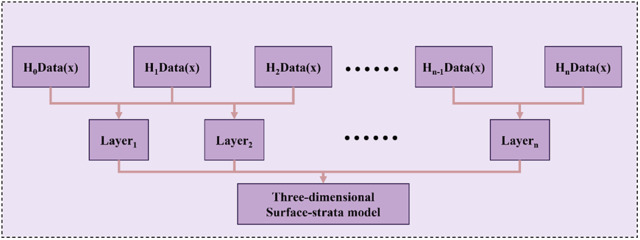
A 3D surface-strata fusion model is established by layering.

In accordance with the absolute elevation of the surface, the absolute elevation of each sequence stratum, and the layer top elevation and layer bottom elevation of each sequence stratum, we employed the Delaunay triangular meshing to establish the 3D surface–stratum fusion model in layers, as illustrated in Fig 4.

### Boolean-difference set operations for 3D surface-stratigraphic-subterranean structure models utilizing the enveloping-box method

In the 3D surface–stratigraphic fusion modeling process, the integration of 3D surface and subsurface data often occurs without considering subsurface structural data. Similarly, in the modeling of subsurface structures, only subsurface structural data are typically prioritized, neglecting surface and subsurface data. However, in reality, surfaces, strata, and subsurface structures are nested together and interact with each other, forming an integrated surface–stratum–subsurface-structure system. Therefore, this paper adopts Boolean-difference set operations based on the enveloping-box method for the geometric fusion of a 3D surface–stratigraphic model and a subsurface-structure model.

**Fig 5:**
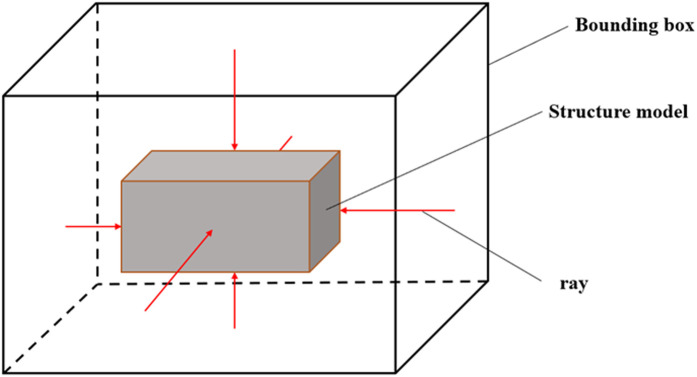
Schematic illustration of enveloping-box method.

The Boolean-difference set operation based on the enveloping-box method begins by extracting the maximum outer contour of the 3D subsurface-structure model. The 3D subsurface-structure model is comprised of numerous components. Involving all components in the difference calculation with the 3D surface–stratum model would result in a significant computational burden and potentially inaccurate final results. Hence, we propose extracting the maximum outer contour boundary of the underground structure using the enveloping-box method, as schematically illustrated in Fig 5.

The specific steps of the enveloping-box method are as follows:

(1) Iterate through the subsurface-structure geometry point data and extract the minimum and maximum points in the XY, YZ, and XZ planes.
(2) Create a sufficiently large enclosing box so that it can enclose the minimum and maximum points of each plane.
(3) Employ the six planes enclosing the box as projection surfaces, and project all geometric point data in the structure onto the projection surfaces to obtain the projected point set, i.e.,P_1_,P_2_,P_3_,P_4_,P_5_,and P_6_.
(4) Create ray sets using each projection point in each projection point set as the source point and the normal direction to the respective projection plane as the ray direction, i.e., Ray_1_,Ray_2_,Ray_3_,Ray_4_,Ray_5_,and Ray_6_.
(5) Extract the initial intersection point of each ray with the subsurface structure model and create a set of intersection points P_intersect_.
(6) Based on the intersection set P_intersect_, fit the outer contour model Model* structure.

**Fig 6:**
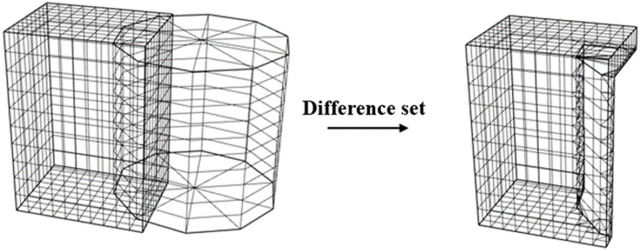
Boolean-difference set operation.

After extracting the maximum outer contour of the subsurface-structure model, a Boolean difference set operation is performed. This operation, grounded in Boolean algebra, involves subtracting one geometric entity from another to ascertain the non-overlapping region between the two. This process is illustrated in Fig 6.

### Attribute fusion method for 3D surface-stratigraphic-subterranean structural models based on variable storage

**Fig 7:**
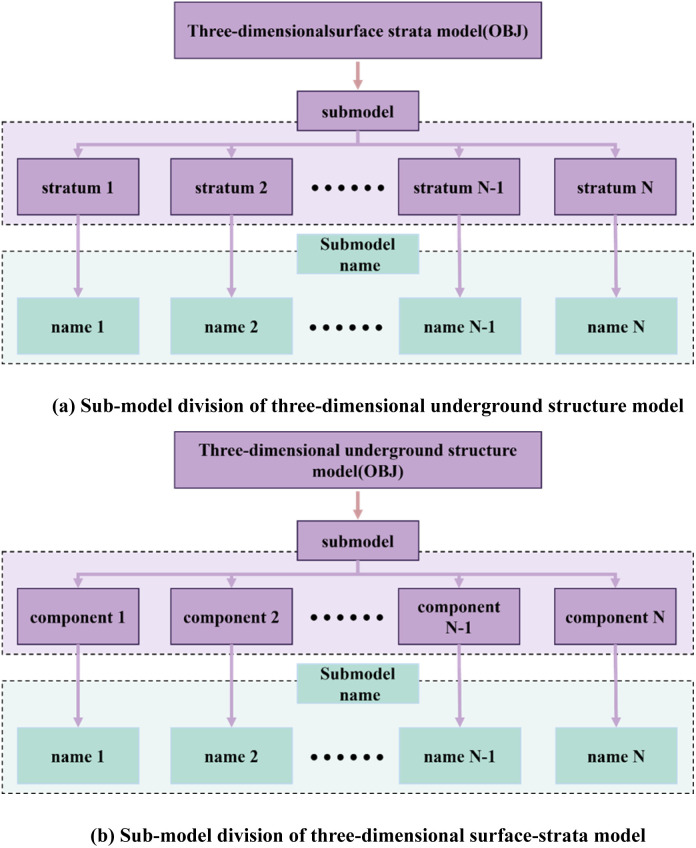
Submodeling of 3D surface–stratigraphic and subsurface-structure models.

Utilizing information-modeling technology, the underground structural model developed in Revit software is typically exported in IFC format, although it can be directly exported to Rvt format files. Its source file contains redundant information data such as the axial network, annotations, etc., in addition to the essential information required for model construction. The attribute data is not shareable with the 3D surface–stratigraphic model and necessitates transformation into intermediate data files of alternate formats to achieve fusion loading between the surface–stratigraphic and underground-structure models. Consequently, the 3D subsurface-structure model must undergo decomposition into the corresponding geometric and attribute data. The spatial and geometric information of the 3D subsurface-structure model is stored in OBJ format (a text-based 3D model file format that defines vertices, faces, and normals), while its attribute information is archived in a JSON text file (a lightweight data interchange format).Furthermore, the OBJ model file is segmented into submodels according to the constituents of the subsurface structure, as illustrated in Fig 7(a).

Analogously, based on the 3D stratigraphic and surface data, the 3D surface–stratigraphic model constructed via the relative-elevation Kriging-interpolation method stores its spatial geometric information in OBJ format. Meanwhile, the JSON text file stores the attribute information of the 3D surface–stratigraphic model and segments the OBJ model file into submodels based on stratigraphic divisions, as illustrated in Fig 7(b).

**Fig 8:**
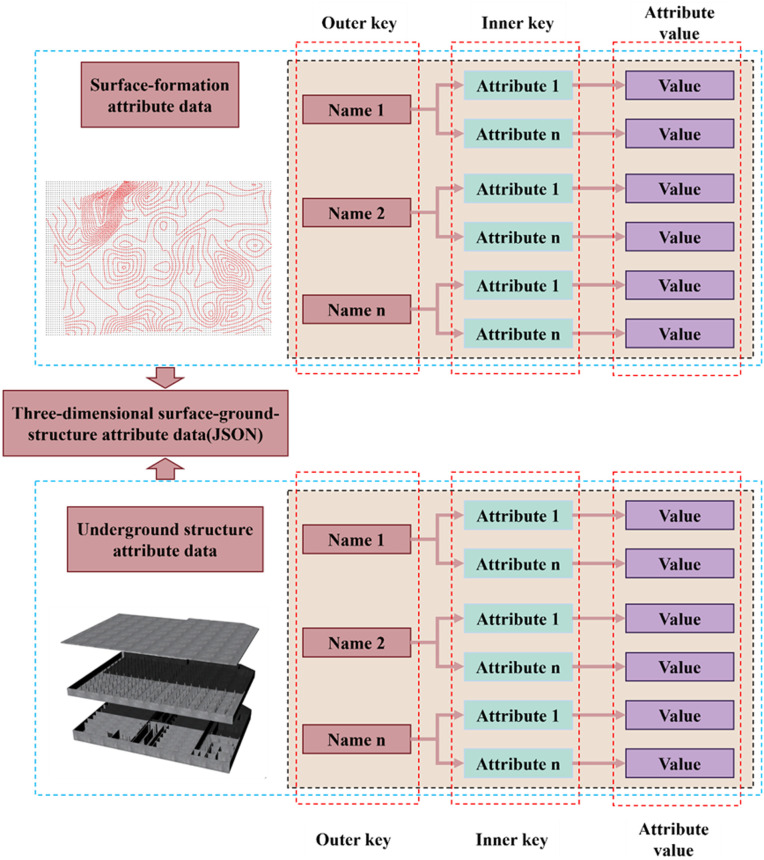
JSON attribute file for 3D surface–stratigraphic–structure models.

To establish a coherent linkage between the geometric and attribute data, the key values in the outer layer of the JSON attribute file are designated as the submodel names within the OBJ model. Meanwhile, the key values in the inner layer signify the required attribute information, with the corresponding attribute values beingstored in the attribute section. The JSON attribute-file storage format of the 3D surface–stratigraphy–structure model is illustrated in Fig 8.

In the context of the web, the JavaScript (JS) language facilitates the definition of array variables through the use of the JS array data type. These variables serve to store the attribute data for 3D surface–stratigraphy–structure models. A salient feature of JS array variables is their capacity to store data in the form of key-value pairs(a fundamental data structure where each unique identifier or “key” is associated with a corresponding “value”). Consequently, the JSON attribute data file of 3D surface–stratigraphy–structure models can be read and parsed, and the attribute data can be mapped into the JS array variables. Additionally, a database is formed to store 3D surface–stratigraphy–structure attribute data and functions are used to call them in real time.

### Dynamic representation of 3D surface–stratigraphic–subterranean structure models

**Fig 9:**
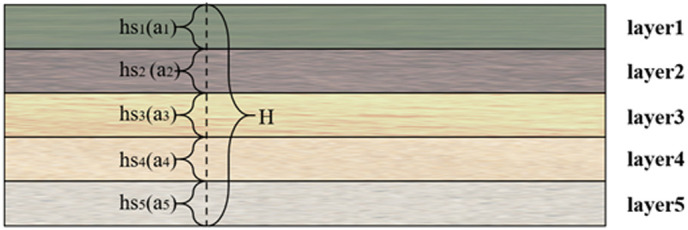
Schematic illustration of monitoring points corresponding to underground spaces without underground structures.

3D dynamic models offer multiple benefits that contribute positively to engineering design, analysis, planning, and decision-making. Widely utilized 3D dynamic models include 3D subsidence dynamic, 3D landslide dynamic, and 3D seismic models. Unlike static representations, dynamic modeling is essential for capturing the time-dependent evolution of geological systems, where mechanical properties and stress conditions continuously change. In this study, we employ the 3D subsidence dynamic model as a paradigmatic example to derive the dynamic representation of 3D models, leveraging the characteristics of a 3D surface–stratum–underground structure model. In the context of the 3D surface–stratum–underground structure model, the underground space corresponding to a monitoring point contains multiple strata or underground-structure components. Consequently, to dynamically characterize the predicted value of stratum settlement within the surface–stratum–underground structure model, it is imperative to ascertain the settlement value of each stratum based on the total stratum-settlement value. This dynamic allocation process is critical because different strata exhibit time-variant compression behaviors under sustained loading. Additionally, the compression coefficient of soil is a crucial mechanical parameter that quantifies the compressive deformation of a soil body under external stresses. It can directly reveal the deformation characteristics of the soil body when subjected to stress. Therefore, the compression coefficient and thickness of the strata can be used in conjunction to determine the weight of each stratum relative to the total surface-settlement value. The settlement value can then be allocated based on the weight. Through this dynamic weighting approach, our model achieves more accurate simulation of stratified settlement progression compared to conventional static methods. An illustration of monitoring points corresponding to underground spaces without underground structures is illustrated in Fig 9.

In Fig 9, “hs” denotes the thickness of the corresponding stratum, “a” denotes the compression coefficient of the corresponding stratum, and “H” denotes the total thickness of all strata. In the absence of subterranean structures within the underground space corresponding to the monitoring point and under the assumption that the given stratigraphic model encompasses “n” strata, the settlement of the ith stratum can be calculated using the thickness of the stratum and the compression coefficient. The calculation employs the following Equation 7:


$$\begin{array}{c} h_{i}={hs}_{i} \end{array}$$ (7)

$$\begin{array}{c} s_{i}=\frac{h_{i}\times a_{i}}{\sum_{m=1}^{n}{(h}_{m}\times a_{m})}\times S \end{array}$$ (8)

In Equation 8, “s_i_” denotes the settlement of the ith stratum, “a_i_” denotes the compression coefficient of stratum i, and “h_i_” denotes the thickness of the stratum in which the settlement occurs in layer i. Additionally, the thickness of the stratum involved in the settlement calculation is equal to the thickness of each stratum, and “S” denotes the total settlement.

**Fig 10:**
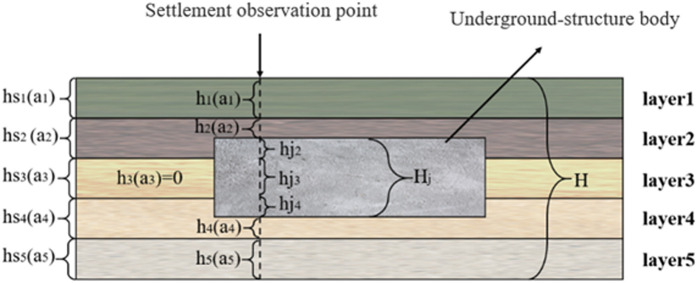
Schematic illustration of monitoring points corresponding to underground spaces with underground structures.

Monitoring points corresponding to underground spaces with underground structures are illustrated in Fig 10.

In Fig 10, “H_j_” denotes the total thickness of the underground-structure body, “h_j_” denotes the thickness of the underground structure in each stratum, and “h” denotes the thickness of the stratum in which settlement occurs. The settlement deformation of soil is predominantly attributed to the alteration in the pore structure of soil when the soil body is exposed to external stress, which causes soil settlement. In contrast, the underground structure is generally a reinforced-concrete structure. In the case of a reinforced-concrete underground structure, its elastic modulus is greater, and the deformation caused by the same external stress is significantly smaller compared to that of the soil body. Therefore, the deformation of the underground structure is deemed negligible when soil settlement occurs. When the soil settles, the deformation of the underground structure is negligible. In cases where the underground space corresponding to the monitoring point contains underground structures, and given the assumption that the stratigraphic model contains n strata, the total thickness of the strata where settlement is observed, H^*^, is expressed as:


$$\begin{array}{c} H^{*}=H-H_{j} \end{array}$$ (9)

The thickness of the stratum in which the subsidence of stratum i occurs, h_i_, is expressed as:


$$\begin{array}{c} h_{i}={hs}_{i}-{hj}_{i} \end{array}$$ (10)

At this time, the thickness h of the stratum involved in the settlement calculation is not equal to the thickness hs of each stratum. Therefore, the thickness h_j_ of the underground structure in each stratum should be subtracted based on Equation 10. Subsequently, h should be substituted into Equation 8 to calculate the settlement of each stratum.s_i_.

In geological investigation reports, the compression modulus of each stratum is commonly provided without the compression coefficient. In such instances, the compression modulus can be converted using the following formula:


$$\begin{array}{c} a=\frac{1+e_{0}}{E_{S}} \end{array}$$ (11)

In Equation 11, a is the compression coefficient, E_S_ the compression modulus, and e_0_ the initial pore ratio.

**Fig 11:**
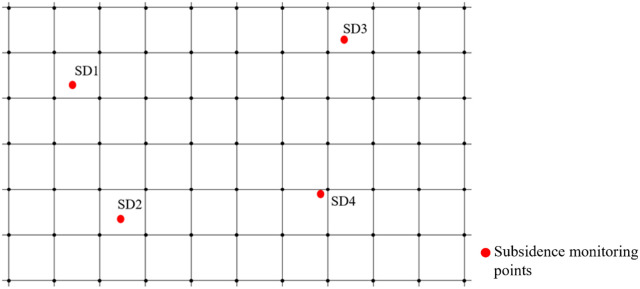
Unified grid network.

For surface–stratigraphic fusion modeling using relative elevation-based Kriging interpolation, a uniform raster grid, Grid_0_, was developed. The relative elevations of each stratum were then interpolated, as illustrated in Fig 11.

Among them, SD1, SD2, SD3, and SD4 correspond to settlement monitoring points. In the raster network Grid_0_, each raster point contains the calculation results of Kriging interpolation based on relative elevation. That is to say, each raster point contains the relative elevation of each stratum beneath the point, as well as the stratum thickness h_s_. To facilitate the dynamic representation of settlement data within a 3D model, it is necessary to allocate the settlement data of the settlement monitoring points (SD1, SD2, SD3, and SD4) to each raster point through an appropriate assignment process. At this juncture, each grid point contains the corresponding stratum thickness h_s_ and the corresponding settlement amount S. Utilizing Equation 8, we can ascertain the settlement amount for each stratum at each grid point. Furthermore, the dynamic stratum thickness h_s_ can be derived by subtracting the corresponding stratum settlement amount s from the stratum thickness h_s_ according to the temporal sequence.

### Hierarchical rendering of 3D surface–stratum–subterranean structure models

**Fig 12:**
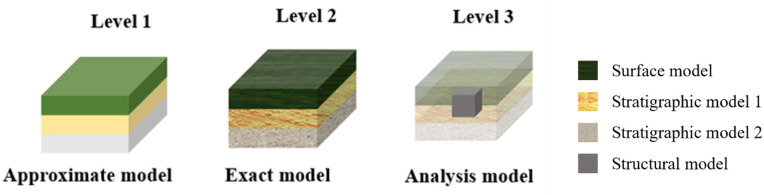
Division of rendering layers.

The lightweight loading method of the 3D surface–stratigraphic–structure model primarily adopts layered-rendering (LOD) technology. The fundamental principle of this technique entails the division of layers based on the observer’s distance or the dimensions of the 3D surface–stratigraphy–structure model displayed on the screen. LOD operates on two key principles: (1) geometric simplification – reducing polygon complexity for distant objects while preserving high-detail meshes for close-range viewing, and (2) texture optimization – dynamically adjusting texture resolution based on visibility requirements. This approach ensures an optimal visual experience for the 3D surface–stratigraphy–structure model, facilitates dynamic adjustments to the model’s level of detail, and reduces the computation and rendering demands while maintaining visual fidelity. Based on the characteristics of the 3D surface–stratigraphy–structure model, the rendering layers are divided as illustrated in Fig 12.

(1) In the approximate model stage, the approximate 3D surface–stratum static model is loaded, along with the basic colors of the model, so as to show the stratigraphic stratification. Conversely, the underground-structure model is not loaded due to its obscurity. In comparison with the case of specific texture mapping, the base color features a smaller data volume and smaller texture size. The base material in the model loading engine contains the base color. Consequently, the external mapping does not need to be loaded for complex texture mapping and computation, thereby enhancing the rendering efficiency and rendering performance of the 3D surface–stratigraphy–structure model.
(2) In the accurate model stage, the precise 3D surface–stratigraphic static model is loaded, along with model-specific texture mapping to accurately represent the stratigraphic layering. Conversely, the subsurface-structure model is not loaded due to its obscurity.
(3) At the model-analysis stage, where the viewpoint approaches the outer surface of the model or infiltrates within it, a 3D surface–stratum–structure model is presented. The pertinent texture maps are then loaded, and the surface–stratum component is rendered translucent to afford a clearer visualization of the 3D subsurface–structure model.

## Results and discussion

To validate the feasibility of the proposed establishment- and lightweight-loading methods for the 3D surface–stratum–underground structure dynamics, we applied the 3D surface–stratum–underground structure dynamic model in a practical engineering context and rendered it in layered detail.

### Project overview

This project in question is a water-purification plant in South China that entails fully buried construction. Following leveling, the ground elevation reached 16m. The plant’s footprint measures 144.2 m × 100.2 m, with the pit bottom ranging in elevation from 1.65 m to 4.65 m. The pit-excavation depth varied between 11 m and 14 m, and the underground structure featured two basement floors. The geological survey work adopts a comprehensive survey method combining drilling, sampling, indoor test, in-situ test, hydrogeological test and other exploration methods. The borehole coordinates and elevation are measured by GNSS-RTK according to coordinates. A total of 88 boreholes were drilled for the project, totaling 2291.60 m in footage and averaging an exploration depth of 26.96 m. The primary stratigraphic lithologies at the site area included: Plain fill, widely distributed in the site area and exposed in all land boreholes, ranging from 0.7 m to 4.0 m in thickness with an average of 1.76 m; Silt, widely distributed in the site area, ranging from 0.5 m to 1.8 m in thickness, with an average of 0.87 m; Pulverulent clay, widely distributed in the site area, ranging from 0.5 m to 1.8 m in thickness, with an average of 0.87 m; Powdery clay, widely distributed in the site and exposed in all boreholes, ranging from 0.5 m to 5.5 m in thickness, with an average thickness of 3.57 m; Medium-coarse sand, distributed in most sections of the site, ranging from 0.4 m to 5.4 m in thickness, with an average of 1.85 m; and grey sandstone, widely distributed in the site and exposed in all boreholes, ranging from 0.2 m to 18.32 m in thickness, with an average of 5.09 m.

### Construction of 3D surface–stratum–subsurface structure dynamic models

(1) Calculation of 3D absolute-surface elevation.

**Fig 13:**
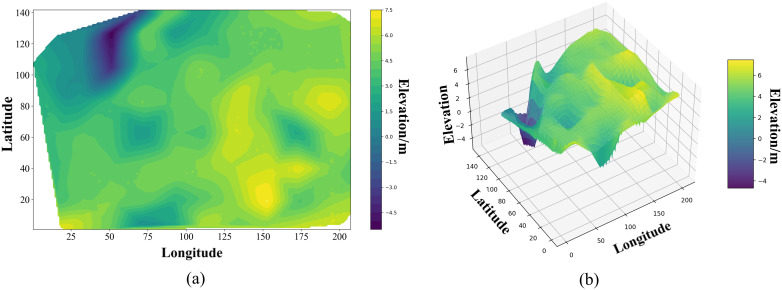
Conversion from contour data to contour point data.

The surface data employed in this case study are surface contour data, as illustrated in Fig 13(a). To more effectively interpolate the surface data in the unified raster grid Grid_0_, it is necessary to transform the surface contour data into spatial contour point data. The selection of the appropriate point distance for converting the surface contour data into spatial contour point data was based on the density of the borehole points, as illustrated in Fig 13(b).

**Fig 14:**
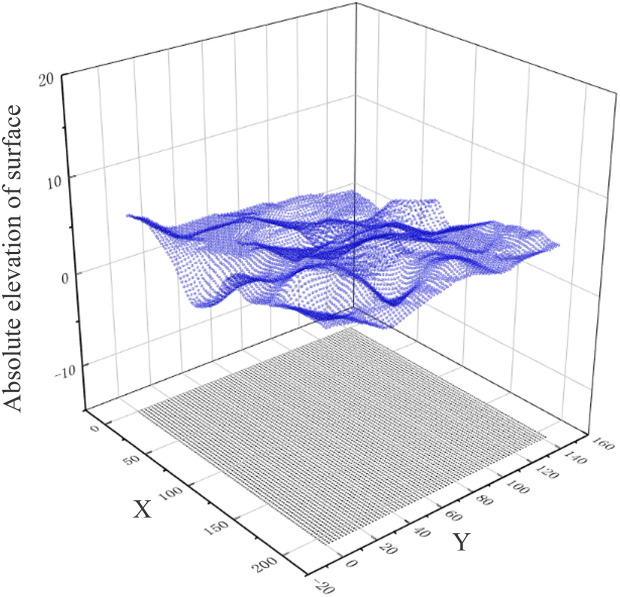
Calculation results of surface absolute-elevation interpolation.

The established unified raster network Grid_0_ contained 72 × 105 = 7560 grid points. Subsequently, utilizing the surface spatial contour point data, a Kriging-interpolation calculation was conducted to derive the absolute-elevation interpolation results for the surface points, denoted as H* 0(x)(x ∈ [1,7560]). The interpolated absolute-elevation values for these surface points are illustrated in Fig 14.

(2) Calculation of relative elevation of underground strata.

The subsurface data utilized in this case study are borehole data, and 88 boreholes were completed. Based on the drilling results, the site was primarily composed of the following stratigraphic layers: (a) vegetative fill layer; (b) silt layer; (c) pulverized clay layer; (d) medium-coarse sand layer; and (e) grey rock layer. To avoid erroneous connectivity between boreholes due to anomalies such as missing, inverted, and duplicated stratigraphy, a unification of the stratigraphic sequence was performed as follows [49]:


$$\begin{array}{c} Seq=[\begin{array}{ccc} ZK1 & ZK2 & \begin{array}{cc} \cdots & ZK88 \end{array} \end{array}]=[\begin{array}{c} \begin{array}{c} 1\\ \begin{array}{c} 2\\ -3 \end{array} \end{array}\\ \begin{array}{c} \begin{array}{c} -4\\ 3 \end{array}\\ \begin{array}{c} 4\\ 5 \end{array} \end{array} \end{array}] \end{array}$$ (12)

Based on Equation 12, the stratigraphic sequence is defined as [1, 2, −3, −4, 3, 4, 5], where strata −3 and −4 represent duplicate occurrences of stratum 3, which is a silty-clay layer, and stratum 4, which is a medium-coarse sand layer, respectively.

Subsequently, the exploration data for each stratum were collated. Additionally, given that the surface data of the case study exclusively pertain to the plain fill of stratum 1, there is no need to delineate the surface data into distinct areas. Therefore, the relative elevation of each stratum can be directly calculated as follows:


$$\begin{array}{c} \left[\begin{array}{c} \begin{array}{c} h_{1}(x)\\ \begin{array}{c} h_{2}(x)\\ h_{-3}(x) \end{array} \end{array}\\ \begin{array}{c} \begin{array}{c} h_{-4}(x)\\ h_{3}(x) \end{array}\\ \begin{array}{c} h_{4}(x)\\ h_{5}(x) \end{array} \end{array} \end{array}]=[\begin{array}{c} \begin{array}{c} H_{0}(x)\\ \begin{array}{c} H_{1}(x)\\ H_{2}(x) \end{array} \end{array}\\ \begin{array}{c} \begin{array}{c} H_{-3}(x)\\ H_{-4}(x) \end{array}\\ \begin{array}{c} H_{3}(x)\\ H_{4}(x) \end{array} \end{array} \end{array}]-[\begin{array}{c} \begin{array}{c} H_{1}(x)\\ \begin{array}{c} H_{2}(x)\\ H_{-3}(x) \end{array} \end{array}\\ \begin{array}{c} \begin{array}{c} H_{-4}(x)\\ H_{3}(x) \end{array}\\ \begin{array}{c} H_{4}(x)\\ H_{5}(x) \end{array} \end{array} \end{array}\right] \end{array}$$ (13)

In Equation 13, h_i_(x) denotes the relative elevation of the xth borehole with stratigraphic sequence i, while H_i_(x) denotes the layer-bottom elevation of the xth borehole within the same stratigraphic sequence i. Specifically, H_o_(x) denotes the surface absolute elevation of the xth borehole.

To ascertain the relative elevation of stratigraphic sequence 1, h_1_(x)(x ∈ [1,88]), we conducted Kriging interpolation utilizing the uniform raster grid Grid_0_. Subsequently, the absolute-elevation (absolute elevation at the bottom of the layer) interpolation result for stratum sequence 1 was calculated based on the interpolated surface absolute-elevation results and the relative-elevation interpolation result of stratum sequence 1, denoted as H* 1(x)(x ∈ [1,7560]).


$$\begin{array}{c} H_{1}^{*}(x)=H_{0}^{*}(x)-h_{1}^{*}(x)\left(x\in [1,7560]\right) \end{array}$$ (14)

**Fig 15:**
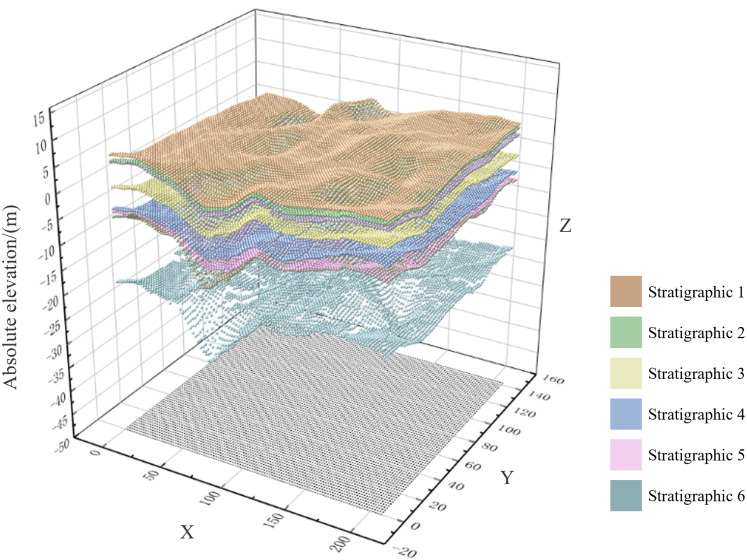
Absolute-elevation settlement results for each stratigraphic sequence.

Similarly, the residual stratigraphic sequences were converted from absolute elevation data to relative elevation data in the exploratory dataset, employing the forementioned methodology. Thereafter, Kriging interpolation was executed based on the relative elevation. Subsequently, the results of the interpolation calculation were converted to absolute elevation. The results of the absolute-elevation settlement for each stratigraphic sequence are illustrated in Fig 15.

In geostatistical analysis, Kriging interpolation is a widely used method for spatial prediction that relies on the modeling of spatial autocorrelation through the semi-variogram. The choice of the semi-variogram model directly impacts the accuracy and reliability of the interpolation results. In this study, we selected an exponential model for its suitability in capturing continuous geological variations within our dataset. The mathematical form of the exponential model is given by:


$$\begin{array}{c} \begin{array}{c} \gamma (h)=\left\{ \;\;\;\begin{array}{c} \;\;0,\;h=0\\ c_{0}+c\left(1-e^{-\frac{h}{a}}\right),h>0 \end{array}\right.\; \end{array} \end{array}$$ (15)

Where, γ(h) is the semi-variance at lag distance, $c_{0}\;$represents nugget effect, c represents partial sill.

(3) Fitting 3D surface–stratigraphic geometric model.

**Fig 16:**
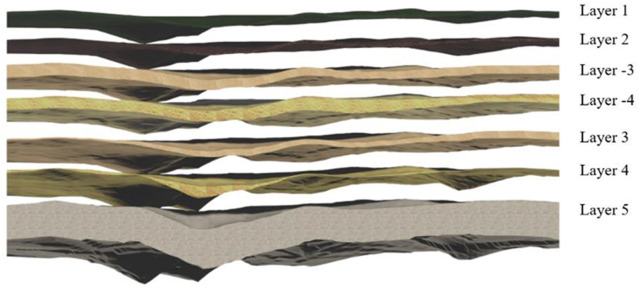
Rendering effect of surface–stratigraphic model.

Utilizing the Kriging-interpolation algorithm grounded in the relative elevation data, we derived eight groups of absolute-elevation calculations, i.e., the absolute-elevation calculation results for the ground surface (H* 0(x)), stratum sequence 1 (H* 1(x)), stratum sequence 2 (H* 2(x)), stratum sequence −3 (H* -3(x)), stratum sequence −4 (H* -4(x)), stratum sequence 3 (H* 3(x)), stratum sequence 4 (H* 4(x)), and stratum sequence 5 (H* 5(x)). H* 0(x) and H* 1(x) represent the layer top and layer bottom elevations of stratum 1, respectively. The layer top and layer bottom of stratum 1 were fitted with H* 1(x), layer top and layer bottom of stratum 1 were fitted with H* 2(x), and so forth. Subsequently, the top and bottom of stratum 5 were fitted with the top and bottom elevations of stratum 5, respectively, and the top and bottom of stratum 5 were interconnected via bridging. The length of the connecting line represents the relative elevation of the corresponding stratum. The appropriate mapping was selected, and the resultant rendering of the model is illustrated in Fig 16.

To achieve optimal geometric congruence with the subsurface structures, the 3D surface–stratigraphic geometry model was stored in an OBJ file.

(4) Geometric fusion of 3D surface–stratigraphic model with 3D subsurface–structure model.

**Fig 17:**
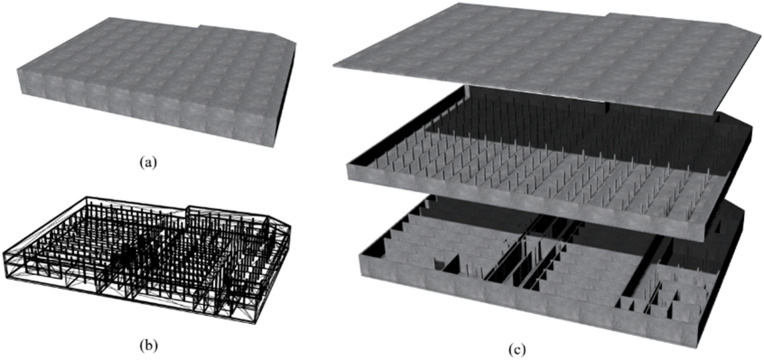
3D underground-structure model.

The underground-structure section of the case study was a two-story basement. The Revit software was used to establish the basement model, and the geometric data of the model were extracted and stored into an OBJ file. The resulting 3D underground-structure model is illustrated in Fig 17.

Fig 17(a), 17(b), and 17(c) show the rendering, model mesh, and internal details of the underground structure, respectively.

**Fig 18:**
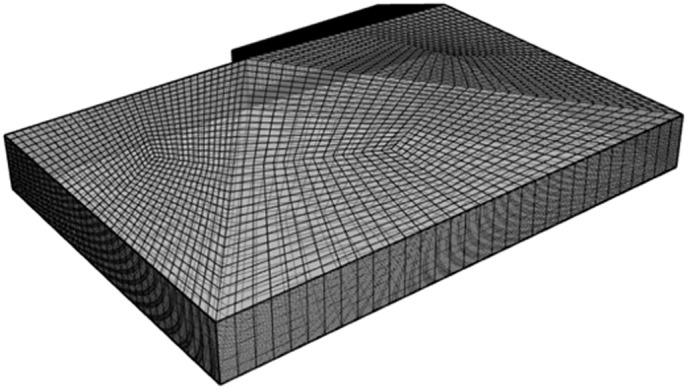
Outer contour grid of underground structure.

To fuse the surface–stratigraphic and subsurface–structure models geometrically, it is first necessary to extract the outer contour model of the subsurface structure using the enveloping-box method, as illustrated in Fig 18.

**Fig 19:**
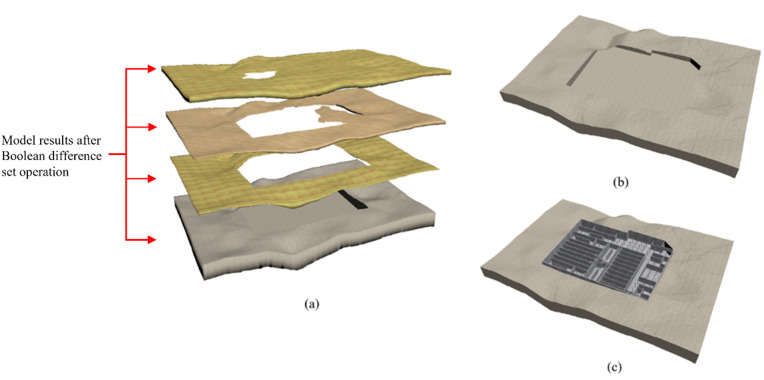
Results of Boolean-difference set operation.

Subsequently, the Boolean-difference set operation was conducted on the outer contour model of the underground structure as the cropping model and on the 3D surface–stratigraphy model as the cropped model. The result of this operation is illustrated in Fig 19.

Fig 16(a) presents the Boolean-operation results for the four strata in the 3D surface–stratigraphic model that intersect with the 3D subsurface–structure model. Fig 16(b) presents the Boolean-operation results of the 3D stratigraphic model for stratigraphic sequence 5, and Fig 16(c) presents the impact of geometric fusion on the 3D surface–stratigraphic–structure model.

(5) Construction of 3D surface–stratum–subterranean structure settlement dynamic model.

**Fig 20:**
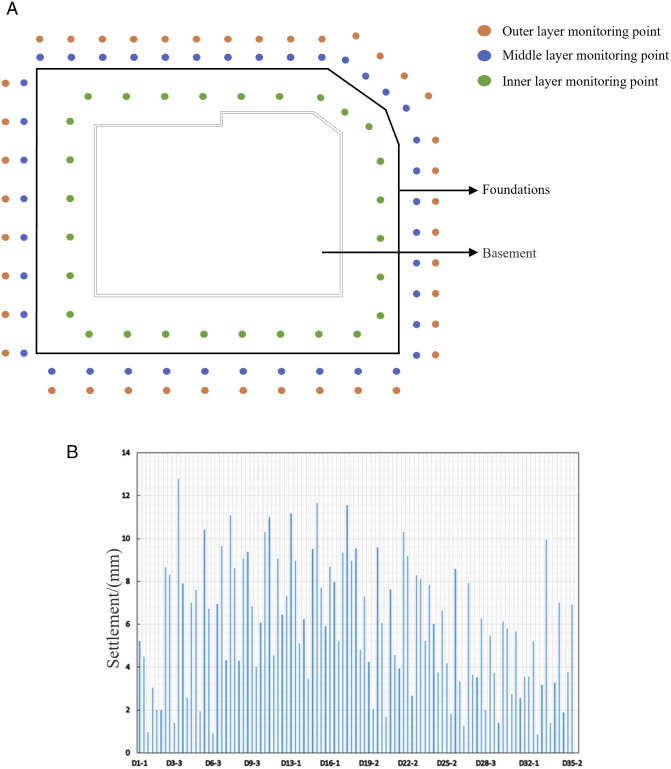
Cumulative monitored sedimentation at each monitoring site.

The project settlement was monitored in accordance with pertinent measurement specifications, encompassing technical directives and the commissioning party’s pit-monitoring program. This monitoring was conducted across the total number of stations, utilizing level meters, hydrometers, and other specialized monitoring instruments. Throughout the duration of pit construction, ground settlement was continuously assessed at 72 monitoring points, with a cumulative total of 5605 monitoring instances executed. Each monitoring point underwent an average of 78 monitoring periods. The cumulative settlement recorded at each monitoring point is illustrated in Fig 20.

The settlement values of 72 monitoring points S(x) were obtained. Additionally, through interpolation calculations, the settlement values of each grid point on the uniform grid network Grid_0_, S*(x)(x ∈ [1,7560]) were derived. The compression modulus of each stratum, as detailed in a geological survey report, is presented in Table 1.

**Table 1:** Compression modulus for each stratum.

Geological sequence	Stratigraphic name	Natural porosity ratio e_0_	Compression modulus E_s_ (0.1–0.2)
1	stockpile soil	0.852	4.0 MPa
2	ooze	0.661	2.0 MPa
−3	silty clay	0.584	6.5 MPa
−4	medium-hard sand	0.334	50 MPa
3	silty clay	0.584	6.5 MPa
4	medium-hard sand	0.334	50 MPa
5	limestone	/	/

Given that the compression modulus of greywacke ranges from 10 to 30 GPa, which is considerably higher than that of other strata, i.e., the compression coefficient is much lower than that of other strata, its settlement value is negligible in comparison. The compression coefficient of each stratum, as determined using the conversion formula between the compression coefficient and compression modulus, is presented in Table 2.

**Table 2:** Compression coefficients for each stratum.

Geological sequence	Stratigraphic name	Compression factor
1	stockpile soil	0.463
2	ooze	0.831
−3	silty clay	0.285
−4	medium-hard sand	0.027
3	silty clay	0.285
4	medium-hard sand	0.027
5	limestone	/

For instance, considering grid point 1, the settlement value on grid point 1S^* (1) is 9.32 mm, and the thickness of each stratum is presented in Table 3:

**Table 3:** Thickness of each stratum.

Geological sequence	Stratigraphic name	Thickness (relative elevation), mm
1	stockpile soil	990
2	ooze	370
−3	silty clay	4260
−4	medium-hard sand	4290
3	silty clay	4210
4	medium-hard sand	1350
5	limestone	10710

Given that the underground space corresponding to grid point 1 lacks any underground structures, it is permissible to directly incorporate this point into Equation 8 for the following calculations:


$$\left\{ \begin{array}{c} {s_{1}}^{*}(1)=\frac{h_{1}\times a_{1}}{h_{1}a_{1}+h_{2}a_{2}+h_{-3}a_{-3}+h_{-4}a_{-4}{+h}_{3}a_{3}+h_{4}a_{4}+h_{5}a_{5}}\times S^{*}(1)\\ \begin{array}{c} \begin{array}{c} \begin{array}{c} {s_{2}}^{*}(1)=\frac{h_{2}\times a_{2}}{h_{1}a_{1}+h_{2}a_{2}+h_{-3}a_{-3}+h_{-4}a_{-4}{+h}_{3}a_{3}+h_{4}a_{4}+h_{5}a_{5}}\times S^{*}(1)\\ {s_{-3}}^{*}(1)=\frac{h_{-3}\times a_{-3}}{h_{1}a_{1}+h_{2}a_{2}+h_{-3}a_{-3}+h_{-4}a_{-4}{+h}_{3}a_{3}+h_{4}a_{4}+h_{5}a_{5}}\times S^{*}(1) \end{array}\\ {s_{-4}}^{*}(1)=\frac{h_{-4}\times a_{-4}}{h_{1}a_{1}+h_{2}a_{2}+h_{-3}a_{-3}+h_{-4}a_{-4}{+h}_{3}a_{3}+h_{4}a_{4}+h_{5}a_{5}}\times S^{*}(1) \end{array}\\ \begin{array}{c} {s_{3}}^{*}(1)=\frac{h_{3}\times a_{3}}{h_{1}a_{1}+h_{2}a_{2}+h_{-3}a_{-3}+h_{-4}a_{-4}{+h}_{3}a_{3}+h_{4}a_{4}+h_{5}a_{5}}\times S^{*}(1)\\ \begin{array}{c} {s_{4}}^{*}(1)=\frac{h_{4}\times a_{4}}{h_{1}a_{1}+h_{2}a_{2}+h_{-3}a_{-3}+h_{-4}a_{-4}{+h}_{3}a_{3}+h_{4}a_{4}+h_{5}a_{5}}\times S^{*}(1)\\ {s_{5}}^{*}(1)=\frac{h_{5}\times a_{5}}{h_{1}a_{1}+h_{2}a_{2}+h_{-3}a_{-3}+h_{-4}a_{-4}{+h}_{3}a_{3}+h_{4}a_{4}+h_{5}a_{5}}\times S^{*}(1) \end{array} \end{array} \end{array} \end{array}\right.\;$$

The calculation results are presented in Table 4.

**Table 4:** Settlement values and post-settlement thickness of each stratum.

Geological sequence	Stratigraphic name	Sedimentation value, mm	Thickness after settlement, mm
1	stockpile soil	1.28	988.72
2	ooze	0.86	369.14
−3	silty clay	3.39	4256.61
−4	medium-hard sand	0.32	4289.68
3	silty clay	3.36	4206.64
4	medium-hard sand	0.1	1349.9
5	limestone	0	10710

Similarly, the stratigraphic settlement values for all remaining grid points were calculated using the aforementioned methodology, yielding the stratigraphic settlement values, denoted as S* i(x) (x ∈ [1,7560])(where i represents the stratigraphic sequence number), as the 3D surface–stratigraphic–structural fusion model was constructed based on the Kriging-interpolation method for relative elevation and featured a unified raster grid Grid_0_. Consequently, the thickness of each stratum (relative elevation) along the connecting line was directly represented in chronological order. Additionally, the settlement thickness could be specified for updating the thickness of each stratum (relative elevation) along the connecting line, enabling the dynamic representation of settlement data in the 3D model.

### Lightweight-loading analysis of 3D surface–stratigraphic–subterranean structure models

The underground-structure model for this case study was constructed using information-modeling technology with the Revit software, which utilizes the Rvt file format. This file format is native to the software and is characterized by its relatively large file size. By extracting the geometric information from the underground-structure file in Rvt format, refining its expression, and eliminating redundant points and surfaces, the model is lightweighted and then stored in an OBJ file format. The specific data obtained after this optimization process are presented in Table 5.

**Table 5:** Memory size of underground structures in different formats.

Model category	File format	File size (Mb)	Optimization rate (%)
Underground structures	Rvt	14.14	0
Underground structures	OBJ	1.42	90

Additionally, the rendering hierarchy was stratified into three broad levels: approximate level (Level 1), accurate level (Level 2), and analytical level (Level 3), based on the distance of the viewpoint from the 3D surface–stratigraphic–structure model.

**Fig 21:**
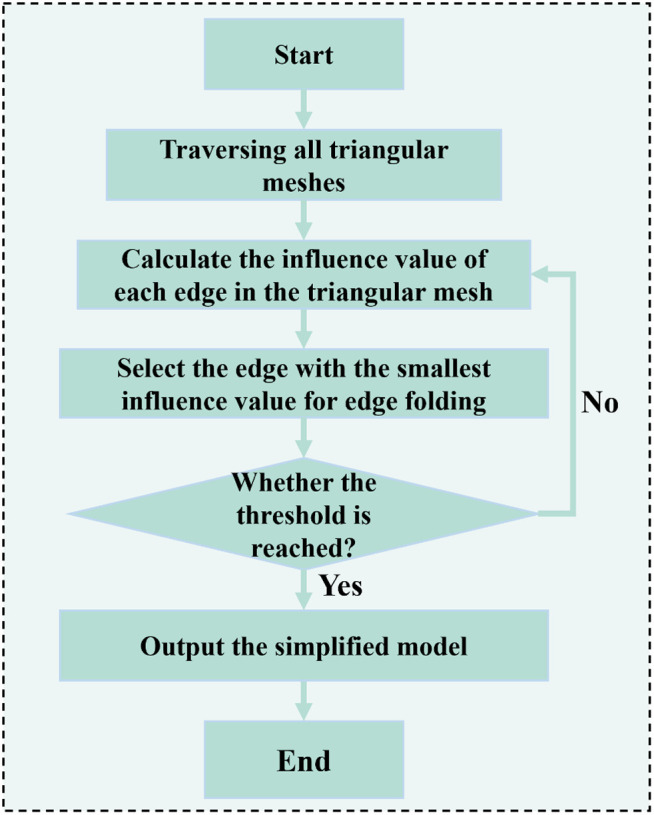
Flow chart of edge folding algorithm.

Subsequently, based on the division of rendering levels and the necessity for loading 3D surface–stratigraphic–structure models at each level. The model is simplified into several 3D surface-strata-structure models by the folding method based on the QEM algorithm. The basic idea of the edge folding algorithm is to calculate the influence of each edge on the 3D surface-strata-structure model after shrinking into points. Select the edge that minimizes the overall impact of the 3D surface-strata-structure model, shrink it into a vertex, and perform multiple iterations. When the number of deleted triangles reaches the preset threshold, terminate the iteration. The algorithm flow is shown in Fig 21.The model was simplified into a number of 3D surface–stratigraphic–structure models. Simplified model 1−1 represents the model rendered at level 1−1, and so on, as illustrated in Table 6.

**Table 6:** Number of triangular surfaces for each simplified model.

Model	Number of triangular surfaces	Simplification ratio (%)
Simplified model 1−1	350	98
Simplified model 1–2	630	97
Simplified model 1–3	1694	91
Simplified model 2−1	3164	85
Simplified model 2−2	6342	70
Original model	21154	0

To enhance the accuracy of the 3D surface–stratigraphic–structure model representation and improve loading efficiency, the approximate level (Level 1) was further divided into three subrendering levels, i.e., Level 1–1 loaded a simplified model 1–1 with 350 triangular facets, level 1–2 loaded simplified a model 1–2 with 630 triangular facets, and Level 1–3 loaded a simplified model 1–3 with 1694 triangular facets. The model maps of these three sublevels were derived from the built-in color maps of the rendering engine.

Furthermore, the accurate level (Level 2) was also subdivided into two subrendering levels, i.e., Level 2−1 loaded a simplified model 2−1 with 3164 triangular facets, while Level 2−2 loaded a simplified model 2−2 with 6342 triangular facets. The model maps of these two subrendering levels were based on specific lithology texture mapping.

**Fig 22:**
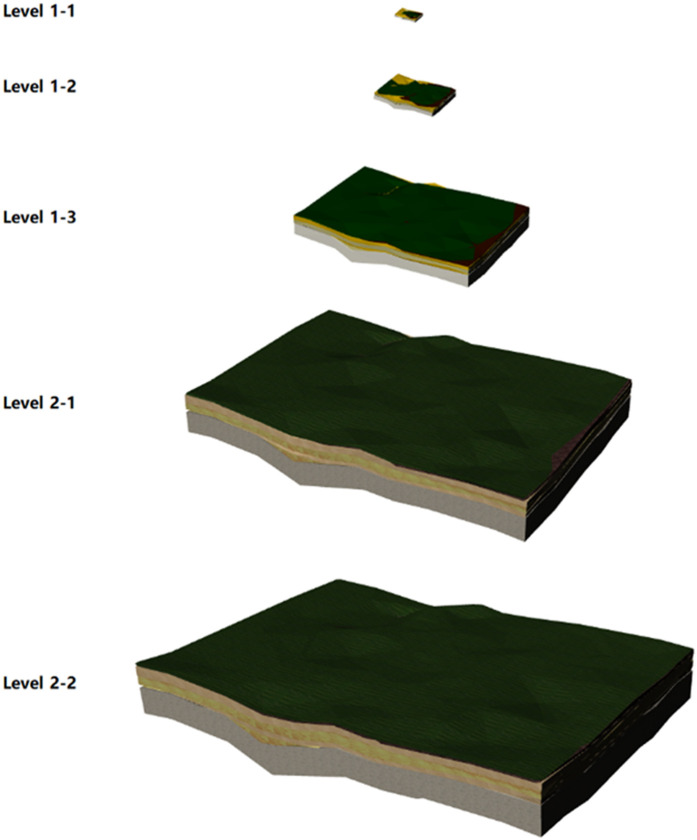
Rendering effect of layers in Levels 1 and 2.

The hierarchical rendering of Levels 1 and 2 is depicted in Fig 22.

**Fig 23:**
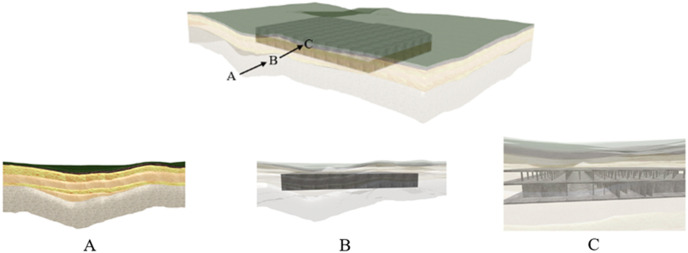
Layer rendering effect in Level 3.

At Level 3, the distance between the viewpoint and the 3D subsurface–structure model is minimal; however, the subsurface–structure model will not be visible owing to the occlusion caused by the 3D surface–stratigraphy model. Therefore, the model must be rendered transparent. Level 3 was subdivided into two rendering sublevels, as illustrated in Fig 23. Points A, B, and C were situated in levels 2−2, 3−1, and 3−2, respectively. In sublevel 3−1, the viewpoint was in close proximity to the surface–stratum model, and the basement model was discernible after the surface–stratum model was rendered transparent. In sublevel 3−2, the viewpoint was in close proximity to the basement façade, and the latter was discernible after rendering it transparent. In sublevel 3−2, the viewpoint was in close proximity to the basement facade, and the internal distribution of the basement was discernible after rendering the basement-facade model transparent.

The rendering efficiency of the 3D surface–stratigraphy–structure model can be quantified by measuring the loading elapsed time, as the loading rate of this model directly affects user experience. The 3D surface–stratigraphy–structure model at each level was tested multiple times, and the average values were recorded. The results of these time-consumption tests are presented in Table 7.

**Table 7:** Average loading elapsed time for each hierarchical model.

Modeling level	Number of triangular surfaces	Average loading time for geometric model, ms	Average total model and material loading time, ms	Total elapsed time optimization rate, %
Level 3	21154	101.3	171.1	0
Level 2−2	6342	57.9	71.5	58
Level 2−1	3164	54.1	65.2	62
Level 1–3	1694	43.5	57.4	66
Level 1–2	630	37.0	44.7	74
Level 1−1	350	35.0	41.8	76

The time-consumption results indicate that Levels 1 and 2 exhibited superior optimization results compared to Level 3. Specifically, the optimization rates for Levels 2 and 1 exceeded 58% and 74%, respectively, resulting in a significant reduction in the loading time of the 3D surface–stratigraphic–structure model.

**Fig 24:**
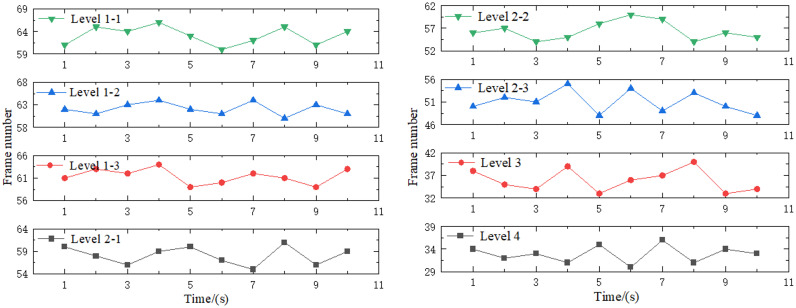
Frame rate test results.

Additionally, the frame rate serves as the most direct indicator of the rendering efficiency of the 3D surface–stratigraphic–structure model. Based on the upper limit of the human body’s capture speed for animation, a frame rate of 30 fps or more ensures smoother model rendering [50]. This can be reflected directly using the upper limit of the human capture speed for animation. The rendering test was conducted on each layer with a frame count of 10 seconds, and the test results are depicted in Fig 24.

The frame rate test results revealed the following frame rates for the levels: Level 3, 33–40 fps; Level 2−2, 54–60 fps; Level 2−1, 55–61 fps; Level 1−3, 59–64 fps; Level 1−2, 60–64 fps; and Level 1−1, 60–66 fps.

### Discussion on research results

Kriging interpolation (including the exponential model used in this study) performs well in the spatial prediction of continuous stratigraphic parameters, but its application has certain limitations: this method assumes that the spatial variability is stable and continuous, so when there are significant discontinuous interfaces such as faults and lithologic mutation zones, the interpolation results may be excessively smooth; in addition, when the sample data is sparse or anisotropic, the reliability of the variogram estimation will be reduced.

## Conclusions

(1) A 3D surface-strata model based on relative elevation Kriging interpolation method is developed, which effectively solves the problem of high-precision surface data and low-precision underground data fusion. The envelope box Boolean difference operation algorithm is proposed to realize the seamless geometric fusion method of surface-stratum-underground structureThen, according to the characteristics of the 3D surface-stratum-underground structure model, the dynamic expression of the settlement data in the 3D model is determined. Then, LOD technology is applied to divide the rendering layer to realize the lightweight loading of the fusion model. Based on the unique characteristics of the 3D surface-strata-underground structure model, the dynamic representation of the settlement data in the 3D model is derived. Then, LOD technology is used to layer the rendering level, so as to realize the efficient and lightweight loading of the fusion model.
(2) This study takes a practical project in southern China as a case to test the effectiveness of the proposed 3D surface-strata-underground structure dynamic model method: by eliminating redundant data and optimizing data expression methods, the model data memory optimization rate reaches 90%; and with the help of the edge folding algorithm of QEM, the maximum simplification rate of a single model is 99.9%. According to the characteristics of the model, LOD grading rendering is performed, and the model loading rate is increased by 58% −76%.
(3) This method is not only applicable to the field of geology and geotechnical engineering, but also can be applied to complex geological modeling, three-dimensional visualization analysis of oil and gas reservoirs, oil pipeline engineering and other engineering scenarios in the development of mineral resources.
